# Adolescent cognitive control processing is associated with anxiety in young adulthood during the COVID-19 pandemic

**DOI:** 10.3758/s13415-025-01293-1

**Published:** 2025-05-19

**Authors:** Grace M. Stohr, Anita Harrewijn, Santiago Morales, Selin Zeytinoglu, Zoë E. Laky, Parmis Khosravi, Elise M. Cardinale, Nicole Lorenzo, Joel Stoddard, Daniel S. Pine, Nathan A. Fox, Melissa A. Brotman, Simone P. Haller

**Affiliations:** 1 Emotion and Development Branch, National Institute of Mental Health, National Institutes of Health, Bethesda, MD USA; 2 Department of Psychology, Education & Child Studies, Erasmus University Rotterdam, Rotterdam, the Netherlands; 3 Department of Psychology, University of Southern California, Los Angeles, CA USA; 4 Department of Human Development and Quantitative Methodology, University of Maryland, College Park, MD USA; 5 Neuroscience and Cognitive Science Program, University of Maryland, College Park, MD USA; 6 Department of Psychology, The Catholic University of America, Washington, DC USA; 7 Psychology Department, American University, Washington, DC USA; 8 Pediatric Mental Health Institute, Children’S Hospital Colorado, Department of Psychiatry & Neuroscience Program, University of Colorado, Anschutz Medical Campus, Aurora, CO USA

**Keywords:** COVID-19 pandemic, FMRI, Adolescents, Anxiety, Stress, Cognitive control

## Abstract

The COVID-19 pandemic has been associated with widespread increases in levels of stress and anxiety among young adults. Given that responses to stressful life events vary, it is important to understand how pre-pandemic neurocognitive factors shape reactivity to stress and susceptibility to anxiety. The present study examines associations between pre-pandemic brain activation patterns during cognitive control processing and anxiety trajectories during the pandemic. Participants were recruited as part of an ongoing longitudinal study of temperament and socioemotional development (*N* = 291). Forty-seven participants completed a cognitive control fMRI task and anxiety measures in late adolescence before the pandemic. In young adulthood, anxiety was assessed three times during the COVID-19 pandemic. Multivariate whole-brain models tested whether activation patterns during the conflict and error processing associated with latent anxiety indices derived from a latent growth curve model. Neural response during conflict and error processing related to anxiety in distinct cortical and subcortical regions. Level of anterior cingulate cortex engagement during cognitive control related to anxiety. However, during error processing, level of engagement in the dorsolateral prefrontal, rather than anterior cingulate cortex, related to anxiety. This work provides preliminary evidence for the predictive utility of prestress neurocognitive factors for young adults’ anxiety response during a uniquely stressful event. Adolescence is a critical time for early identification of youth at risk to create targeted interventions to enhance stress resilience.

**Supplementary information:**

The online version contains supplementary material available at 10.3758/s13415-025-01293-1.

## Introduction

The COVID-19 pandemic involved increased anxiety for many people, especially young adults on the precipice of autonomy (Barendse et al., 2021; Stoddard et al., 2023). Heterogeneity in anxiety outcomes during the pandemic may relate to the degree of stress experienced during the pandemic and patterns of risk present before pandemic onset (Cabello-Toscano et al., 2023; Loades et al., 2020; Morales et al., 2021, 2022; Zeytinoglu et al., 2021). Establishing temporal precedence can highlight how prestress neurocognitive factors shape young people’s vulnerability to stress-related anxiety symptoms. Thus, the current study leverages longitudinal data to examine how pre-pandemic cognitive control and associated neural functions relate to young adults’ anxiety during the pandemic.

Cognitive control supports flexible, adaptive behavior through component processes, including the ability to select, implement, and update behavior to meet one’s goals (Egner, 2023; Miyake & Friedman, 2012; Miyake et al., 2000). Aberrant cognitive control, specifically in the ability to inhibit prepotent responses and monitor errors, relates to concurrent anxiety (Dudeney et al., 2015; Fani et al., 2012; Hayes et al., 2012; MacLeod & Mathews, 1988; McTeague et al., 2017). However, inconsistency exists in the directionality of these associations; some studies find enhanced cognitive control functions in people with relatively high levels of anxiety (Buzzell et al., 2021; Filippi et al., 2020; Meyer et al., 2017; Smith et al., 2020; Valadez et al., 2021), whereas other studies find the reverse (Derakshan & Eysenck, 2009; Eysenck et al., 2007). These inconsistencies may be in part due to unique associations between specific components of cognitive control (Cardinale et al., 2023) and phenotypes of anxiety (Smith et al., 2020). This highlights the need to examine both specific subdomains of cognitive control and specific expressions of anxiety.

Few longitudinal studies examine relationships between cognitive control subdomains and particular expressions of anxiety (Cardinale et al., 2023; Drexler et al., 2024). Generally, cognitive control and associated activation patterns mature across adolescence (Bunge et al., 2002; Crone & Steinbeis, 2017; Davidson et al., 2006; Ernst & Mueller, 2008; Luna et al., 2001; Rubia et al., 2006). Relatively high levels of cognitive control ability may confer resilience to stress by facilitating effective management of the source of stress and adaptive coping strategies (De Lissnyder et al., 2012; Hammar et al., 2023; Hoorelbeke et al., 2015; Wu et al., 2021). However, data on the prospective relationship between the neural correlates of conflict processing and anxiety in the context of stress are lacking.

Previous electrophysiological and functional magnetic resonance imaging (fMRI) studies have implicated the cingulate cortex and lateral prefrontal cortex as well as the insula, basal ganglia, and thalamus, in error monitoring (Buzzell et al., 2017; Tamnes et al., 2013; Velanova et al., 2008). Other work links error-related indices to cortisol response (Compton et al., 2013) and reports of negative affect to daily stressors (Compton et al., 2011). The most relevant study (Morales et al., 2021), examined the same cohort as the current study, quantified the error-related negativity—an event-related potential component reflecting rapid response to errors in early/mid-adolescence. Higher levels of responding to errors and a more reactive control profile predicted higher levels of anxiety during the first few months of the pandemic.

Using the same cohort, the current study considers whether other cognitive control indices, specifically conflict and error processing, assessed using fMRI in late adolescence relate to anxiety during the pandemic. Given the inconsistencies in prior work, the directionality of the expected associations remains unclear.

## Methods

### Participants

Participants were youth and their caregivers, recruited from an ongoing longitudinal study of temperament and socioemotional development (2001 to present; *N* = 291; 53.6% female). The study followed children recruited at 4 months of age based on evoked positive and negative emotional and motor reactivity to novel stimuli (Hane et al., 2008). Maternal racial and ethnic identity reported by the mother at the time of enrollment was primarily non-Hispanic Caucasian (69.4%), followed by African American (16.5%), Hispanic (7.2%), Asian (3.1%), other (3.4%), and missing (0.3%). Maternal education levels were high, with 35.7% graduate degrees, 41.9% college graduates, 16.2% high school graduates, 5.5% receiving other forms of education, and 0.7% missing information. Of the initial sample of 291 participants, a subset of 162 participants completed the first online anxiety assessment between April 20 and May 15 of 2020, which was approximately 1 month (*M* = 29.67, *SD* = 6.01 days, *M*_age_ = 18.20, *SD*_age_ = 0.66) after the stay-at-home order was implemented in the state of residence of most participants. A second assessment was completed by 153 participants approximately 1 month later (*M* = 26.48, *SD* = 7.31 days) when business and public spaces gradually reopened. A final assessment included 144 participants 1 month later (*M* = 28.86, SD = 5.83 days) after stay-at-home orders were lifted and businesses had reopened.

Of the 162 participants, 48 completed a cognitive control task in the scanner, of whom 47 participants also completed an anxiety questionnaire at the time of the fMRI scan. Their data were used in the analyses below. On average, the fMRI scan visit and the first pandemic anxiety assessment were ~2 years (*M* = 1.75, *SD* = .77 years) apart; participants were ~16 years (*M*_age_ = 16.41, *SD*_age_ = 0.45 years) at the time of the fMRI scan and ~18 years (*M*_age_ = 18.17, *SD*_age_ = 0.64) at the time of the first pandemic anxiety assessment. A summary of demographic characteristics as self-reported by the participant at age 18 years is shown in Table 1. A comparison of demographic and clinical characteristics between participants who completed the fMRI task and those who did not can be found in the supplementary materials. No significant differences emerged between the two samples; the fMRI sample was trending towards lower anxiety at the first assessment.

**Table 1:** Demographic characteristics and questionnaire data from 47 participants

	Scan participants (n = 47)	
	*n (%)*	*M (SD)*
Sex	47	
Females	22 (46.81)	
Males	25 (53.19)	
Age at scanning time point	47	16.41 (0.45)
Race	47	
American Indian/Alaskan Native	32 (64.385)	
Asian/Pacific Islander	1 (2.17)	
Black	7 (15.22)	
More than one race	1 (2.17)	
White	35 (76.09)	
Ethnicity	42	
Hispanic/Latino/a/x	7 (16.67)	
Not Hispanic/Latino/a/x	35 (83.33)	
Questionnaire assessments		
SCARED at scanning timepoint^a^	47	11.61 (10.24)
Anxiety (GAD-7) T1	45	4.42 (5.54)
Anxiety (GAD-7) T2	46	3.80 (4.62)
Anxiety (GAD-7) T3	43	3.65 (5.21)
Total Worries (COVID-19 Worry Scale) T1	46	2.58 (0.79)
Total Worries (COVID-19 Worry Scale) T2	46	2.35 (0.72)
Total Worries (COVID-19 Worry Scale) T3	44	2.12 (0.65)
Stress (PSS-10) T1	39	17.13 (7.60)
Stress (PSS-10) T2	45	15.96 (6.80)
Stress (PSS-10) T3	44	14.45 (8.69)

*Note*. T1, T2, and T3 indicate the three COVID-19 assessment timepoints. Race and ethnicity were self-reported at age 18. SCARED: The Screen for Child Anxiety-Related Emotional Disorders; PSS-10 = Perceived Stress Scale – 10-item version

^a^Youth- and parent-report SCARED were averaged for the anxiety assessment at the fMRI scan visit

Written informed consent was obtained from caregivers and assent from youth participant before enrollment. Youth older than 18 years provided written informed consent.

### Procedure

At ~16 years, participants from a longitudinal study of temperament and socioemotional development completed a cognitive control fMRI task as well as the SCARED self-report anxiety measure (*n* = 47). Between April 20 and July 2020, the same participants, now aged ~18 years, completed an online measure of generalized anxiety symptoms and stress monthly, up to three times.

### Measures

#### Anxiety

Anxiety at the scanning time point was measured using The Screen for Child Anxiety Related Emotional Disorders (SCARED; Birmaher et al., 1997). The SCARED is a 38-item dual-informant questionnaire that surveys anxiety disorder symptoms occurring within the past 3 months. Items were summed to create a total score (range 0–82). The SCARED has good internal consistency (α = 0.74–0.93) and moderate-to-good test-retest reliability (Child: 0.59–0.61, Parent: 0.74–0.86; Runyon et al., 2018). Youth- and parent-report SCARED were averaged for the anxiety assessment at the fMRI scan visit.

Anxiety during the pandemic was measured using the GAD-7 (Spitzer et al., 2006). The GAD-7 is a 7-item self-report questionnaire that surveys symptoms of generalized anxiety disorder experienced within the past 2 weeks. Items were summed to create a total score (range: 0–21). Scores ≥ 10 suggest clinically significant generalized anxiety. The GAD-7 has excellent internal consistency (α = 0.92) and good test-retest reliability (0.83; Spitzer et al., 2006).

#### Stress

Perceived stress during the pandemic was measured by using the Perceived Stress Scale (PSS-10; Cohen et al., 1983). The PSS-10 consists of 10 items querying levels of experienced stress. The total score, a sum of 10 items, shows good internal consistency, α = 0.89 (Roberti et al., 2006) construct and concurrent validity (Mitchell et al., 2008).

COVID-19–specific stress was assessed by using the COVID-19 worry scale (Morales et al., 2022), an adaptation of the COVID-19 Adolescent Symptom & Psychological Experience Questionnaire (CASPE; Ladouceur, 2020). The self-report questionnaire consists of 18 items to assess concerns about pandemic-related stressors and disruptions. The COVID-19 worry scale showed good test-retest reliability (*r*s > 0.73) and internal consistency (α = 0.88) in data from the same cohort (Morales et al., 2022).

#### fMRI cognitive control task

A modified Eriksen Flanker task (Eriksen & Eriksen, 1974) was used to measure cognitive control. A jittered fixation cross (300–600 ms) preceded five arrows in a single line centered on the screen (200 ms), followed by a blank response screen of 1700 ms. In the congruent trials, all arrows pointed in the same direction, whereas in the incongruent trials, the center arrow pointed in the opposite direction compared with the four adjacent arrows. Participants were instructed to respond to the direction of the center arrow as quickly as possible. Trials were presented at random across four runs of 218 trials per trial type; 120 additional fixation-only trials were randomly interspersed to increase the inter-trial interval. The task was divided into 12 blocks between which participants received feedback based on task accuracy (<75%: “be more accurate”; 75–90%: “good job”; >90%: “respond faster”). Consistent with prior work (Cardinale et al., 2023; Smith et al., 2020), this feedback was included to balance accuracy while maximizing errors.

### Analytic plan

The analytic plan was preregistered (https://osf.io/bmzda^1^). The clinical data have been published previously in a larger sample composition (Lorenzo et al., 2021; Morales et al., 2021, 2022; Zeytinoglu et al., 2021). As noted below, we examine outcomes consistent with previous reports in our subsample with fMRI data.

#### Clinical measures

Consistent with previous research in this sample (Morales et al., 2021), a latent growth curve model was applied to the longitudinal clinical data using the lavaan statistical package version 0.6–17 in R version 4.4.1. The model was fit to the 162 participants who completed clinical assessments during the pandemic. We estimated the latent intercept (indexing anxiety levels at the first assessment) and latent slope (indexing linear change in anxiety across all three assessments). The former was estimated by constraining the paths of each month to 1. The latter by constraining the paths for each month (month 1, month 2, and month 3) to 0, 1, and 2, respectively. A larger sample allows for more accurate estimation of model parameters. Analogous analyses for stress measures and associations between stress and anxiety measures are contained in the supplementary materials.

### Behavior

#### Cognitive control

Mean reaction times (RTs) per condition were calculated and RTs less than 150 ms were removed. Participants with an accuracy of ≥70% were retained in the analysis (*n* = 47). The group level analyses applied separate ANOVAs testing associations between latent intercept and slope and task condition (congruent/incongruent correct trials) with age and anxiety at the scan visit as covariates.

#### Error monitoring

Post-error slowing was calculated as the mean RT following errors compared with the mean RT following correct responses on incongruent trials. Only participants with at least 20 commission errors were included in the analysis (*n* = 43). The group-level analyses applied separate ANOVAs to test associations between latent intercept and slope and task condition (postincongruent correct/incongruent commission errors) with age, anxiety at the scan visit and number of commission errors as covariates.

#### fMRI data acquisition and processing

Functional magnetic resonance imaging data were acquired on a 3-T GE imaging system with a 32-channel head coil. Functional image volumes were collected with an in-plane resolution of 2.5 × 2.5 mm using a T2-weighted gradient-echo pulse sequence (repetition time = 2000 ms, echo time = 25 ms, flip angle = 60°, field of view = 96 × 96, slices = 42/axial/3 mm). A high-resolution 3-dimensional MPRAGE spin-echo sequence (repetition time = 7.66, echo time = 3.42, flip angle = 7°, field of view = 256 × 256, slices = 176/sagittal/1 mm) was also acquired to aid co-registration and normalization procedures.

Data were processed and analyzed by using Analysis of Functional NeuroImages (AFNI, version 24.0.02). The preprocessing included slice timing correction, coregistration, normalization, and nonlinear registration of echoplanar data to anatomical scans. Subsequently, data were smoothed (to target of 6.5-mm full-width-half-maximum, suing a flexible Gaussian kernel and resulting in an average effective smoothness of 9.2), resampled to 2.5-mm isotropic voxels, and intensity scaled to 100. Volumes with more than 10% outliers and TR pairs with a Euclidean norm motion derivative greater than 1 mm were censored.

First-level analyses were conducted by creating a general linear model (GLM) for each participant with each stimulus onset event type entered as separate regressors (congruent and incongruent correct trials, congruent and incongruent commission and omission errors). Additional regressors included baseline drift and motion (rotational movement of roll, pitch, and yaw, and motion displacement in the x, y, and z axes).

Quality control procedures used the APQC (Reynolds et al., 2023) to perform visual inspection of alignment between structural and functional scans and examine a variety of quantitative measures and visual tools to assess image quality. To be included in the analysis, no more than 15% of TRs across conditions could be censored, and average motion could not exceed 0.25 mm after censoring.

#### fMRI group-level analysis

AFNI’s 3dMVM program was used for all group-level analyses. Separate multivariate models tested the associations between the latent intercept and slope and task condition (conflict processing: congruent/incongruent, error processing: commission error/correct in the incongruent condition) was entered as the within-subject independent variable. Age and anxiety at the scan visit were included as covariates. For the error analysis, only youth with ≥20 commission errors were included (*n* = 43), and the number of commission errors was added as an additional covariate for the error analysis.

Whole-brain analyses were conducted within a grey matter mask where at least 90% of participants contributed data. The voxel-wise threshold was set at *p* < .005 with cluster-correction at alpha = .05 (first nearest neighbor), which resulted in a cluster extent of *k* = 53 via Monte Carlo simulation with a gaussian plus exponential spatial autocorrelation function to estimate smoothness (AFNI’s 3dClustSim program). To characterize whole-brain associations, the mean activity for significant clusters was extracted using AFNI’s 3dROIstat program. Because our imaging sample size was modest, data were particularly carefully assessed for influential cases using Cook’s distance. Iterative post-hoc analyses leaving out influential cases (Cook’s distance > 0.5) were done to ensure that findings were robust to their exclusion.

## Results

### Anxiety trajectories, overall sample (*N* = 162)

**Fig. 1:**
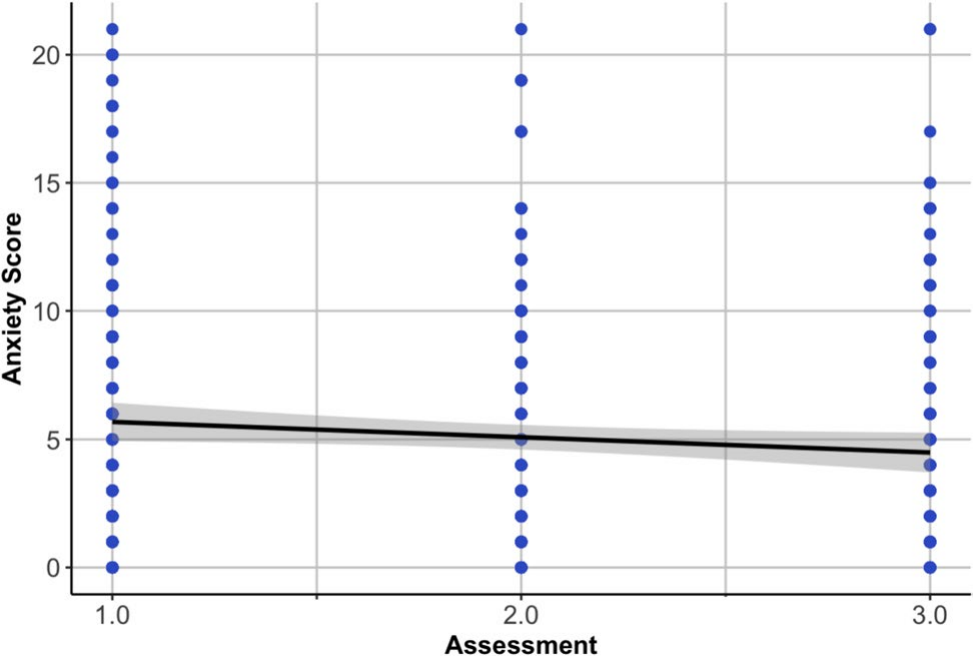
Average trajectory of anxiety and raw total scores (GAD 7-Item Scale) from the first to the third assessment. The growth curve model showed a good fit (χ^2^_1_ = 0.01, *p* = .94, root mean square error of approximation = 0.00, [90% confidence interval 0.00, 0.054], standardized root mean square residual = 0.00, comparative fit index = 1.00), as also reported by (Morales et al., 2021)

As detailed in prior work (Morales et al., 2021, 2020), across the sample of 162 who completed the questionnaires, anxiety gradually decreased across the three measurements (*b* = −0.73, *p* = .001; Fig. 1; see supplementary materials for analogous analyses of stress measures). Clinical levels of anxiety (GAD-7 scores ≥ 10) were experienced by approximately 20.0% of the participants at the first assessment post-pandemic onset, which steadily declined to 18% and 17%, respectively, over the follow-up assessments. Notably, the intercept (anxiety at the first assessment) and slope (trajectory over three assessments) correlated significantly (*r*(160) = −.77, *p* < .001), such that those with a higher initial peak at the first assessment also showed a steeper decline over the subsequent two assessments. For associations between anxiety and stress measures across time points, see supplementary materials.

### Anxiety trajectories, fMRI sample (*n* = 47)

In our subsample that completed the fMRI scan visit at age ~16 years, we found relative stability in anxiety over time between the fMRI scan visit and the first pandemic assessment (*r*(45) = .39, *p* = .01). Analyses focused on two anxiety variables: the latent intercept (anxiety at the first assessment) and slope (linear trajectory over three assessments). Relations between these variables in the subsample were identical to those in the larger sample (*r*(45) = −.77, *p* < .001). Because of the largely shared variance amongst the two indices, latent intercept and slope, we focused on the latent intercept, i.e., anxiety at the first assessment. Because both indices were preregistered, additional analyses for the latent slope can be found in the supplementary materials.

### Behavior

#### Conflict processing

Reaction time differences in congruent and incongruent correctly completed trials did not significantly associate with the latent intercept (*F*(1,43) = .01, *p* = .92, η^2^ < .001).

### Error monitoring

Reaction time differences between trials after a commission error and after a correctly completed incongruent trial did not associate with the latent intercept (*F*(1,39) = .72, *p* = .40, η^2^ = .02).

### Neural activation

For maps of the main effect for each contrast (conflict processing, error monitoring), see the supplementary materials.

#### Neural response to conflict

**Fig. 2:**
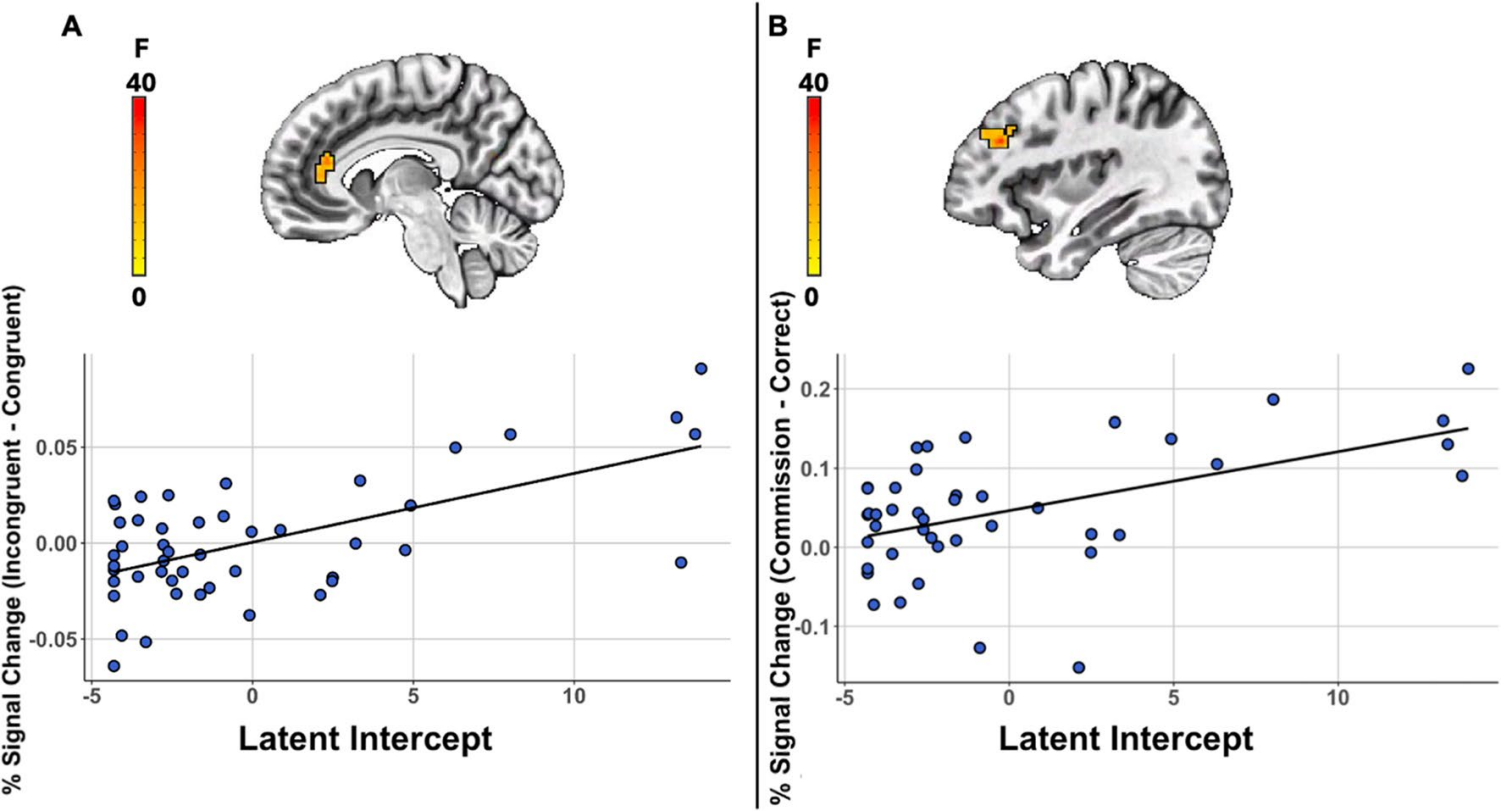
A. Significant associations of neural activity during conflict processing with anxiety symptoms at the first assessment (latent intercept) in the left ACC. **B**. Significant associations of neural activity in the left middle frontal gyrus during error processing with anxiety symptoms at the first assessment

Table 2 details all significant results for the cognitive conflict analysis. Significant intercept-by-condition interactions were observed in five regions, including the left hippocampus (*F*(1,43) = 35.44, *p* < .001, η^2^ = .45, *r*(45) = .63), the left fusiform gyrus (*F*(1,43) = 38.49, *p* < .001, η^2^ = .47, *r*(45) = .59), the right amygdala (*F*(1,43) = 23.32, *p* < .001, η^2^ = .35, *r*(45) = .56), the right middle temporal gyrus (*F*(1,43) = 19.55, *p*<.001, η^2^ = .31, *r*(45) = .52) and left anterior cingulate cortex (*F*(1,43) = 38.32, *p* < .001, η^2^ = .47, *r*(45) = .61; Fig. 2A).

**Table 2:** Significant associations of neural activity during conflict processing with anxiety symptoms at the first assessment (latent intercept)

Region	Cluster Size		Coordinates (center of mass)			Coordinates (at peak)			Mean F	SEM	Max Int	Post-hoc^a^
	k	mm3	CM LR	CM PA	CM IS	MI LR	MI PA	MI IS				
Left Hippocampus, Left Fusiform Gyrus, Left Parahippocampal Gyrus	103	1609.38	−27	−33.1	−9.6	−27.5	−29.2	−7	13.60	0.53	46.23	.63
Left Fusiform Gyrus, Left Cerebellum (VI)	85	1328.13	−33.5	−54.9	−20	−40	−54.2	−22	13.20	0.55	34.27	.59
Right Amygdala	72	1125.00	26.1	−2.6	−15.1	35	−1.8	−17	11.99	0.40	26.76	.56
Right Middle Temporal Gyrus	65	1015.63	54.4	−59.9	13.3	52.5	−59.2	15.5	11.84	0.39	24.37	.52
Left Anterior Cingulate Cortex	53	828.13	−8	34.3	15	−7.5	33.2	15.5	14.87	0.63	27.86	.61

*Note*. Cluster-corrected voxel-wise linear multivariate model results are presented, summarizing regions showing a significant intercept-by-condition interaction. Location lists regions in descending order based on proportion overlap with cluster. k = number of voxels in cluster; mm3 = cluster volume; CM = center of mass of cluster; MI = max intensity (peak); SEM = standard error of the mean; LR = left-right (x); PA = posterior-anterior (y); IS = inferior-superior (z)

^a^Post-hocs are correlation coefficients between the latent intercept and the % signal change in the task contrast

#### Neural response to error

Table 3 details all significant results for the error analysis. Significant intercept-by-error interactions were observed in two regions, the left middle frontal gyrus (*F*(1,38) = 29.24, *p* < .001, η^2^ = .44, *r*(41) = .51; Fig. 2B) and the left middle cingulate cortex/paracentral lobule (*F*(1,38) = 33.93, *p* = .001, η^2^ < .47, *r*(41) = .62).

**Table 3:** Significant associations of neural activity during error processing with anxiety symptoms at the first assessment (latent intercept)

Region	Cluster Size		Coordinates (center of mass)			Coordinates (at peak)			Mean	SEM	Max Int	Post-hoc^a^
Overlap	k	mm3	CM RL	CM AP	CM IS	MI RL	MI AP	MI IS				
Left Middle Frontal Gyrus	88	1375	31.8	−34.7	31.2	35	−33.2	28	14.74	0.55	34.68	.51
Left Middle Cingulate Cortex, Left Paracentral Lobule	64	1000	11.8	29.9	46.8	10	29.2	50.5	12.16	0.46	27.66	.62

*Note*. Cluster-corrected voxel-wise linear multivariate model results are presented here summarizing regions showing a significant intercept-by-condition interaction. Location lists regions in descending order based on proportion overlap with cluster. k = number of voxels in cluster; mm3 = cluster volume; CM = center of mass of cluster; MI = max intensity (peak); SEM = standard error of the mean; LR = left-right (x); PA = posterior-anterior (y); IS = inferior-superior (z)

^a^Post-hocs are correlation coefficients between the latent intercept and the % signal change in the task contrast

## Discussion

Two key findings emerged from this study. First, during the pandemic, an initial peak manifested in anxiety, which was followed by a steady decline, reflecting the intercept and slope from a latent growth curve model. These two parameters strongly correlated: those with relatively high levels of anxiety at the first measurement point also showed relatively steep declines in anxiety over the first 3 months of the pandemic as public life resumed. Second, unlike behavior on the flanker task, neural regions engaged by events requiring cognitive control and error processing manifested relations with later anxiety in distinct regions.

Individuals with higher levels of anxiety at the first assessment also showed a steeper decline of anxiety over the 3 months, parallelling significant events during the first wave of the pandemic. Our data suggest that some individuals were more reactive to the initial events, but also recovered and were able to compensate for the initial increase. It is plausible that this is characteristic of individuals with adaptive coping strategies and significant resources (Asmundson et al., 2020; Lorenzo et al., 2021). Before the pandemic, the youth in this study were free of treatment-requiring psychopathology and likely embedded in functioning social support networks, given their ability to participate in a two-decade long cohort study. The sociodemographics of the current sample were such that youth came from largely moderate-to-high socioeconomic status households; the reported educational attainment for our sample (~77% reported holding a college or graduate degree) was significantly higher than the national average.

Reaction time on the flanker task probing cognitive control at age ~16 did not associate with anxiety during the pandemic, controlling for concurrent anxiety at the scan. However, in the absence of behavioral differences, an increased neural response when exerting cognitive control was positively associated with the initial peak in anxiety, mostly in regions that were not primarily engaged by the task, such as the amygdala and the ACC. The amygdala has been more commonly implicated in tasks requiring threat detection and broad negative emotional processing; however, more recent work suggests a much broader role of this structure in salience processing (Ousdal et al., 2008). For instance, West et al. (2021) demonstrated that better cognitive functioning associated with less amygdala activation during a nonemotional working-memory task.

Associations between activation in regions recruited on average across the sample and anxiety, such as the fusiform gyrus during conflict processing or the dlPFC during error processing, may index a compensatory effort signal aimed at maintaining a standard level of performance. Hence, increased activation in these regions may reflect inefficient cognitive control and greater utilization of processing resources to perform up to par. Inefficient cognitive control may associate with patterns of hypervigilance during the pandemic and therefore confer anxiety vulnerability (Eysenck et al., 2007).

Given the modest sample size, it is important to consider these findings preliminary. Recent work suggests that sample sizes needed to obtain robust estimates of brain-behavior associations are in the thousands (Marek et al., 2022). It is prohibitively challenging to collect a fMRI sample of this size, especially with the goal of studying stress-related anxiety through the lens of a specific process. Longitudinal designs increase power through repeated measurement. In the same realm, collecting more samples (i.e., events) as part of the fMRI protocol can significantly increase statistical efficiency (Chen et al., 2021). Here, we collected repeated measures of anxiety over time for each participant, and for cognitive control contrast analyses specifically, we had many events per task condition (>180 trials). The additional care in phenotyping and sampling hopefully increases robustness of the signal; replication in other contexts, however, will be critical.

### Limitations

Beyond the modest sample size, another limitation of the current study is the change in anxiety measures administered at the time of the scan and during the pandemic (SCARED and GAD-7). Albeit not ideal, it was important to control for concurrent anxiety at the time of the fMRI visit to capture potential increases in anxiety. The generalizability of the current findings is limited by the sample composition—a community sample with a large proportion of white families with educational attainment and income above the national average. Not all individuals and communities have experienced the same impact of the global pandemic. Those already burdened prior to the pandemic have been impacted the hardest. It will be important to ensure cohort studies sample youth that reflect the diversity of the culture and conditions to allow for better generalizability and any benefit of research innovation to be applicable to the population.

## Conclusions

A few studies to date have been able to examine pre-stressor neural patterns (Chahal et al., 2021; Foster et al., 2023; Haller et al., 2024; Hardi et al., 2023; Kitt et al., 2023; Miller et al., 2021; Perica et al., 2021; Sequeira et al., 2021; Weissman et al., 2021), but most studies rely on retrospectively collected data. Epidemiological work has identified adolescence and young adulthood as a key risk period for the emergence of impairing, clinically significant levels of anxiety (Kessler et al., 2012). Hence, adolescence is a critical time for early identification of youth at risk to create targeted interventions to enhance stress resilience. This work provides preliminary evidence for the predictive utility of prestress neurocognitive factors for young adults’ anxiety response during a uniquely stressful event.

## Supplementary information

Below is the link to the electronic supplementary material.

Supplementary file1 (DOCX 678 KB)

## Data Availability

In accordance with the NIH Data Management and Sharing Policy, MRI data relevant to this manuscript will be shared in a repository for those participants that consented to share their data publicly: https://openneuro.org/. Individual difference data collected during COVID-19 was acquired at the University of Maryland. This data with matching identifiers is available upon reasonable request from S.H. or N.A.F. respectively. The data are not publicly available due to privacy restrictions.

## Footnotes

1 The preregistration includes a separate set of analyses on a threat processing task collected at a different scan visit. The analysis of this data has not been completed yet. A smaller number of participants completed the threat processing task.

## Code availability

Analytic code will be made available on GitHub: https://github.com/NIMH-SDAN.

The analytic plan was pre-registered: https://osf.io/bmzda.
