# Supplementary material for: Adolescent cognitive control processing is associated with anxiety in young adulthood during the COVID-19 pandemic
Source: Cogn Affect Behav Neurosci. 2025 May 19;25(5):1485–95. doi: 10.3758/s13415-025-01293-1 (PMC12464013; doi:10.3758/s13415-025-01293-1)
Supplement: Supplementary file 1 — Supplementary file1 (DOCX 678 KB) [file 13415_2025_1293_MOESM1_ESM.docx]

Supplementary Materials

for

# Adolescent cognitive control processing is associated with anxiety in young adulthood during the COVID-19 pandemic

1. **A comparison of demographic characteristics between participants who completed the fMRI task and those who did not undergo a scan**

Table S1. Comparison of demographic characteristics

| **Variable** | **fMRI scan (N=47)** | **No fMRI scan (N=115)** | **Stat** | **p** | **es** |
| --- | --- | --- | --- | --- | --- |
| Age (at first assessment) | 18.17 (0.64) | 18.22 (0.67) | t=0.47 | 0.64 | d=0.08 |
| Anxiety (at first assessment) | 4.42 (5.6) | 6.21 (5.57) | t=1.81 | 0.07 | d=0.32 |
| Sex (female) | 22 (46.81%) | 48 (41.74%) | χ2=0.17 | 0.68 | OR=0.82 |
| Latent Intercept ^t^ | 4.56 (5.2) | 6.39 (5.2) | t=2.03 | 0.05 | d=0.35 |
| Latent Slope | -0.53 (1.14) | -0.81 (1.27) | t=-1.36 | 0.18 | d=-0.23 |
| Race (white) | 35 (79.55%) | 73 (81.11%) | χ2=0.00 | 1.0 | OR=1.10 |
| *Note*. T1, T2, and T3 indicate the three COVID-19 assessment timepoints. N=5 missing Age (at first assessment). N=7 missing Anxiety (at first assessment). N=27 missing Perceived Stress (at first assessment). N=5 missing Total Worries (at first assessment). N=28 missing Race (white). * p<.05, ** p<.01, *** p<.001, ^t^ p<.10 | | | | | |

1. **Stress and worry trajectories**

Stress measured by the PSS-10 gradually decreased across the three measurements (*b* = -2.01, *p* < .001). The growth curve model showed a good fit (χ^2^_1_ = 0.66, p = .42, root mean square error of approximation = 0.00, [90% CI, 0.00, 0.19], standardized root mean square residual = 0.013, comparative fit index = 1.0).

Similarly, COVID-19 worries decreased steadily across the three timepoints (*b* = -0.24, *p* < .001). The growth curve model showed an adequate fit (χ^2^_1_ = 1.83, p = .18, root mean square error of approximation = 0.07, [90% CI, 0.00, 0.24], standardized root mean square residual = 0.018, comparative fit index = 1.0).

1. **Associations between anxiety and stress/worries**

As detailed in prior work (Morales et al., 2022), in the overall sample who completed the questionnaires, anxiety and perceived stress strongly correlated across time points, as did anxiety and COVID-19 worries (see Table S2).

Table S2. Correlations between anxiety and stress measures for the overall sample *N*=162

|  | Anxiety (GAD-7) T1 | Anxiety (GAD-7) T2 | Anxiety (GAD-7) T3 |
| --- | --- | --- | --- |
| Total Worries (COVID-19 Worry Scale) T1 | 0.48*** [155] | .46*** [148] | .48*** [134] |
| Total Worries (COVID-19 Worry Scale) T2 | 0.54*** [146] | .59*** [153] | .52*** [139] |
| Total Worries (COVID-19 Worry Scale) T3 | 0.51*** [133] | .48*** [140] | .53*** [141] |
| Stress (PSS-10) T1 | 0.69*** [135] | .63*** [126] | .50*** [115] |
| Stress (PSS-10) T2 | 0.59*** [142] | .68*** [149] | .48*** [136] |
| Stress (PSS-10) T3 | 0.39*** [136] | .55*** [135] | .73*** [138] |

*Note.* In brackets is the sample size for that measure after listwise deletion.

* indicates *p*<.05, ** indicates *p*<.01,*** indicate *p*<.001

T1, T2, and T3 = first, second, and third assessments during the COVID-19 pandemic

In the subsample that completed the fMRI scan visit at age ~16, we found associations of similar magnitude for anxiety and stress and COVID-19 worries respectively (see Table S3).

Table S3. Correlations between anxiety and stress measures for fMRI subsample *n*=47

|  | Anxiety (GAD-7) T1 | Anxiety (GAD-7) T2 | Anxiety (GAD-7) T3 |
| --- | --- | --- | --- |
| Total Worries (COVID-19 Worry Scale) T1 | 0.57*** [45] | 0.58*** [45] | 0.46** [42] |
| Total Worries (COVID-19 Worry Scale) T2 | 0.56*** [44] | 0.57*** [46] | 0.45** [42] |
| Total Worries (COVID-19 Worry Scale) T3 | 0.49*** [42] | 0.53*** [43] | 0.49*** [43] |
| Stress (PSS-10) T1 | 0.77*** [39] | 0.70*** [38] | 0.59*** [35] |
| Stress (PSS-10) T2 | 0.69*** [43] | 0.70*** [45] | 0.50*** [42] |
| Stress (PSS-10) T3 | 0.71*** [42] | 0.66*** [43] | 0.82*** [43] |

*Note.* In brackets is the sample size for that measure after listwise deletion.

* indicates *p*<.05, ** indicates *p*<.01,*** indicate *p*<.001

T1, T2, and T3 = first, second, and third assessments during the COVID-19 pandemic

1. **Main effect for each task contrast, i.e. cognitive conflict and error processing**

Table S4 and S5 detail all significant results for the task contrasts. Figure S1 panel A/B provide a visual illustration of the task network engaged during conflict and error processing, respectively.

Table S4. Regions showing significant neural activity during conflict processing

| Region | Cluster Size | | Coordinates (center of mass) | | | Coordinates (at peak) | | | Mean F | SEM | Max Int |
| --- | --- | --- | --- | --- | --- | --- | --- | --- | --- | --- | --- |
|  | k | mm3 | CM LR | CM PA | CM IS | MI LR | MI PA | MI IS |  |  |  |
| Right Superior Parietal Lobule, Right Inferior Parietal Lobule | 1794 | 28031.25 | 37.20 | -58.30 | 38.00 | 45.00 | -44.20 | 55.50 | 15.78 | 0.15 | 48.81 |
| Left Inferior Parietal Lobule, Left Superior Parietal Lobule | 718 | 11218.75 | -35.10 | -54.30 | 52.50 | -37.50 | -51.80 | 60.50 | 13.01 | 0.14 | 34.19 |
| Left Inferior Occipital Gyrus, Left Middle Occipital Gyrus | 267 | 4171.88 | -46.50 | -74.70 | -4.10 | -52.50 | -74.20 | -2.00 | 13.28 | 0.22 | 26.85 |
| Cerebellar Vermis (6), Left Cerebellum, Cerebellar Vermis (4/5) | 262 | 4093.75 | -1.10 | -66.80 | -19.20 | -5.00 | -71.80 | -17.00 | 12.64 | 0.22 | 26.15 |
| Right Cerebellum (VI), Right Cerebellum (Crus_1) | 236 | 3687.50 | 28.90 | -56.90 | -26.80 | 32.50 | -59.20 | -32.00 | 12.30 | 0.20 | 24.91 |
| Left Cerebellum (VI), Left Cerebellum (Crus_1) | 225 | 3515.63 | -32.90 | -58.50 | -27.40 | -32.50 | -49.20 | -32.00 | 13.52 | 0.31 | 38.02 |
| Right Insula Lobe | 190 | 2968.75 | 41.60 | 14.80 | 2.60 | 52.50 | 10.80 | -2.00 | 12.33 | 0.23 | 29.98 |
| Right Middle Cingulate Cortex, Right Anterior Cingulate Cortex, Right Superior Medial Gyrus | 178 | 2781.25 | 6.30 | 28.30 | 35.40 | 7.50 | 28.20 | 30.50 | 12.22 | 0.21 | 22.87 |
| Left Insula Lobe | 165 | 2578.13 | -40.00 | 13.90 | 3.10 | -40.00 | 10.80 | 0.50 | 12.69 | 0.25 | 22.73 |
| Left SMA, Right SMA | 119 | 1859.38 | 0.80 | 4.90 | 51.40 | 0.00 | 5.80 | 50.50 | 12.39 | 0.37 | 31.85 |
| Right Middle Frontal Gyrus | 113 | 1765.63 | 30.50 | 47.60 | 32.80 | 27.50 | 48.20 | 35.50 | 14.50 | 0.47 | 30.28 |
| Left Precentral Gyrus | 65 | 1015.63 | -50.70 | 7.70 | 40.20 | -47.50 | 10.80 | 43.00 | 11.38 | 0.25 | 17.92 |

*Note*. Cluster-corrected voxel-wise linear multivariate model results are presented here summarizing regions showing a significant intercept-by-condition interaction. Location lists regions in descending order based on proportion overlap with cluster. k=number of voxels in cluster, mm3=cluster volume, CM=center of mass of cluster, MI=max intensity (peak), SEM=standard error of the mean, LR=left-right (x), PA=posterior-anterior (y), IS=inferior-superior (z).

Table S5. Regions showing significant neural activity during error processing

| Region | Cluster Size | | Coordinates (center of mass) | | | Coordinates (at peak) | | | Mean F | SEM | Max Int |
| --- | --- | --- | --- | --- | --- | --- | --- | --- | --- | --- | --- |
|  | k | mm3 | CM LR | CM PA | CM IS | MI LR | MI PA | MI IS |  |  |  |
| Right Middle Frontal Gyrus, Left Insula | 8213 | 128328.13 | 6.90 | 19.50 | 23.90 | 32.50 | 18.20 | 5.50 | 28.51 | 0.22 | 175.25 |
| Left Thalamus, Right Thalamus | 2066 | 32281.25 | 0.70 | -18.20 | 14.10 | 5.00 | -29.20 | 25.50 | 21.78 | 0.28 | 80.91 |
| Right Inferior Parietal Lobule, Right SupraMarginal Gyrus, Right Angular Gyrus, Right Middle Temporal Gyrus | 2065 | 32265.63 | 50.90 | -44.20 | 36.30 | 62.50 | -41.80 | 35.50 | 20.83 | 0.26 | 98.92 |
| Left Inferior Parietal Lobule, Left Superior Parietal Lobule | 1442 | 22531.25 | -39.80 | -52.70 | 44.00 | -52.50 | -41.80 | 48.00 | 18.98 | 0.26 | 86.55 |
| Left Calcarine Gyrus, Right Calcarine Gyrus | 1261 | 19703.13 | 0.50 | -74.20 | 8.00 | 17.50 | -71.80 | 13.00 | 16.89 | 0.19 | 44.58 |
| Right Putamen, Left Putamen | 711 | 11109.38 | 2.30 | 1.20 | -1.10 | 30.00 | -9.20 | 8.00 | 17.04 | 0.27 | 47.36 |
| Left Middle Frontal Gyrus | 572 | 8937.50 | -33.20 | 45.80 | 23.10 | -35.00 | 43.20 | 25.50 | 19.64 | 0.38 | 58.24 |
| Left Precuneus, Right Precuneus, Left Calcarine Gyrus | 478 | 7468.75 | -4.20 | -58.80 | 18.00 | -10.00 | -56.80 | 15.50 | 19.33 | 0.47 | 59.70 |
| Left Mid Orbital Gyrus, Left Rectal Gyrus, Right Mid Orbital Gyrus | 431 | 6734.38 | -1.10 | 54.30 | -12.10 | 2.50 | 53.20 | -12.00 | 16.55 | 0.33 | 41.13 |
| Right Precuneus, Right Cuneus | 411 | 6421.88 | 11.40 | -70.70 | 46.30 | 10.00 | -74.20 | 48.00 | 14.42 | 0.24 | 28.37 |
| Left Cerebellum (Crus 1), Left Cerebellum (VI) | 320 | 5000.00 | -35.00 | -63.60 | -29.60 | -35.00 | -56.80 | -32.00 | 18.66 | 0.59 | 67.15 |
| Left Middle Occipital Gyrus, Left Angular Gyrus | 249 | 3890.63 | -42.60 | -76.90 | 34.10 | -45.00 | -76.80 | 35.50 | 21.01 | 0.74 | 59.64 |
| Left Middle Frontal Gyrus, Left Superior Frontal Gyrus | 159 | 2484.38 | -22.30 | 27.60 | 45.40 | -17.50 | 28.20 | 45.50 | 13.57 | 0.40 | 36.24 |
| Right ParaHippocampal Gyrus, Right Hippocampus | 148 | 2312.50 | 23.20 | -13.30 | -19.80 | 17.50 | -11.80 | -22.00 | 15.95 | 0.52 | 39.19 |
| Left Hippocampus | 140 | 2187.50 | -22.50 | -12.00 | -19.30 | -20.00 | -11.80 | -19.50 | 20.57 | 1.11 | 83.51 |
| Right Precentral Gyrus, Right Postcental Gyrus | 137 | 2140.63 | 32.60 | -25.00 | 61.30 | 25.00 | -24.20 | 70.50 | 11.85 | 0.23 | 22.29 |
| Left Fusiform Gyrus | 128 | 2000.00 | -32.50 | -40.30 | -13.60 | -32.50 | -39.20 | -12.00 | 20.52 | 1.08 | 76.38 |
| Right Inferior Temporal Gyrus | 125 | 1953.13 | 46.70 | -3.80 | -36.50 | 50.00 | -6.80 | -37.00 | 20.29 | 0.78 | 51.75 |
| Right Cerebellum (Crus 1), Right Cerebellum (VI) | 112 | 1750.00 | 38.30 | -58.30 | -31.50 | 32.50 | -54.20 | -32.00 | 15.43 | 0.50 | 33.69 |
| Right Superior Temporal Gyrus, Right Middle Temporal Gyrus | 111 | 1734.38 | 61.00 | -8.60 | -9.20 | 60.00 | -6.80 | -14.50 | 13.37 | 0.38 | 29.32 |
| Right Fusiform Gyrus, Right ParaHippocampal Gyrus | 105 | 1640.63 | 30.60 | -36.50 | -15.50 | 32.50 | -36.80 | -14.50 | 15.45 | 0.50 | 34.13 |
| Left Inferior Frontal Gyrus | 85 | 1328.13 | -34.50 | 33.20 | -12.10 | -40.00 | 30.80 | -12.00 | 15.67 | 0.65 | 36.21 |
| Right Hippocampus, Right ParaHippocampal Gyrus | 85 | 1328.13 | 21.80 | -33.20 | -4.20 | 22.50 | -34.20 | -7.00 | 13.06 | 0.37 | 23.57 |
| Right SupraMarginal Gyrus, Right Postcentral Gyrus | 80 | 1250.00 | 61.80 | -18.20 | 28.00 | 60.00 | -16.80 | 25.50 | 15.29 | 0.56 | 27.98 |
| Right Inferior Frontal Gyrus | 76 | 1187.50 | 31.90 | 36.00 | -10.00 | 32.50 | 35.80 | -12.00 | 17.09 | 0.68 | 30.36 |
| Left SupraMarginal Gyrus, Left Postcentral Gyrus | 76 | 1187.50 | -63.40 | -22.20 | 29.80 | -65.00 | -21.80 | 28.00 | 12.85 | 0.39 | 22.79 |
| Right Middle Orbital Gyrus, Right Superior Orbital Gyrus | 62 | 968.75 | 23.30 | 54.60 | -14.30 | 27.50 | 58.20 | -14.50 | 12.27 | 0.35 | 21.84 |
| Left ParaHippocampal Gyrus, Left Lingual Gyrus | 57 | 890.63 | -21.20 | -38.90 | -3.70 | -22.50 | -36.80 | -7.00 | 12.68 | 0.49 | 29.50 |
| Left Superior Medial Gyrus, Left Superior Frontal Gyrus | 54 | 843.75 | -9.60 | 68.80 | 18.00 | -10.00 | 68.20 | 20.50 | 12.15 | 0.34 | 19.41 |

*Note*. Cluster-corrected voxel-wise linear multivariate model results are presented here summarizing regions showing a significant intercept-by-condition interaction. Location lists regions in descending order based on proportion overlap with cluster. k=number of voxels in cluster, mm3=cluster volume, CM=center of mass of cluster, MI=max intensity (peak), SEM=standard error of the mean, LR=left-right (x), PA=posterior-anterior (y), IS=inferior-superior (z).


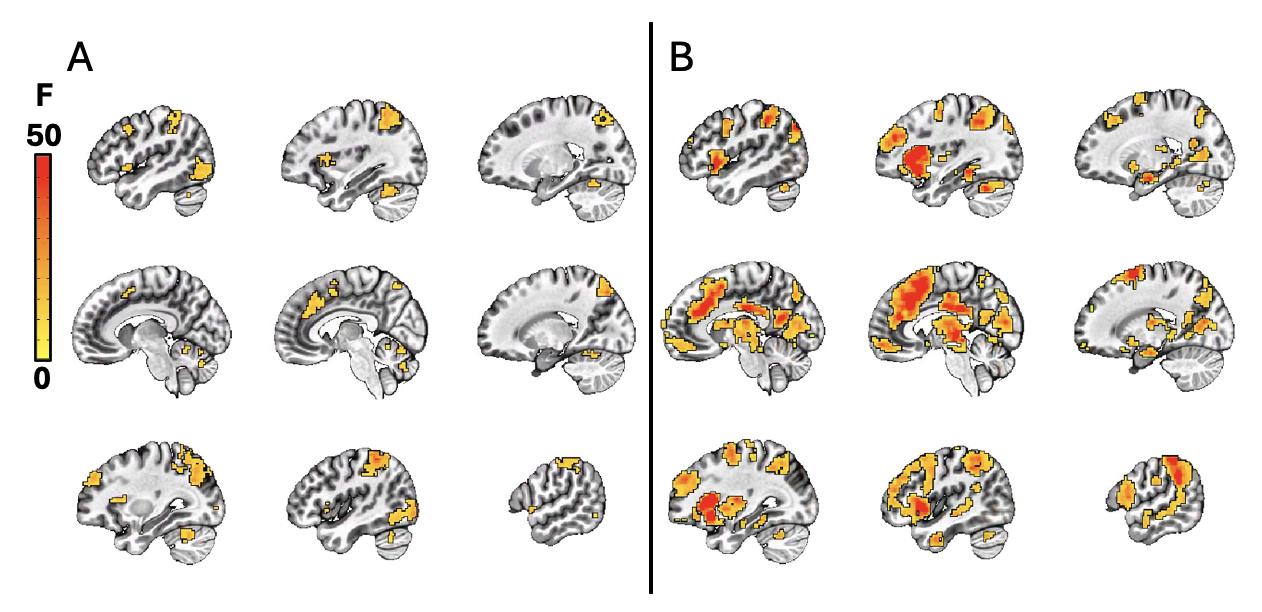


Figure S1. Regions showing significant activation during cognitive conflict and error processing

1. **Behavior**

*Conflict processing*

Reaction time differences in congruent and incongruent correctly completed trials did not significantly associate with the latent intercept (*F*(1,43)=.21, *p*=.65, η^2^=.01).

*Error monitoring*

Reaction time differences between trials after a commission error and after a correctly completed incongruent trial did not associate with the latent intercept (*F*(1,39)=.33, *p*=.57, η^2^=.01).

*Neural Response to Conflict*

Table S6 details all significant results for the cognitive conflict analysis. Significant slope-by-condition interactions were observed in seven regions including the right middle temporal gyrus (*F*(1,43)=27.27, *p*<.001, η^2^<.39, *r*(45)=-.62), the right Rolandic operculum (*F*(1,43)=30.46, *p*<.001, η^2^<.42, *r*(45)=-.62), the right postcentral gyrus (*F*(1,43)=33.96, p<.001, η^2^<.44, *r*(45)=-.65), the right SMA (*F*(1,43)=30.74, *p*<.001, η^2^<.42, *r*(45)=-.64), the left Rolandic operculum (*F*(1,43)=33.03, *p*<.001, η^2^<.43, *r*(45)=-.66), the right Rolandic operculum (*F*(1,43)=30.30, *p*<.001, η^2^<.41, *r*(45)=-.64) and the right supramarginal gyrus (*F*(1,43)=28.74, *p*<.001, η^2^<.40, *r*(45)=-.61).

Table S6. Significant associations of neural activity during cognitive conflict processing with anxiety symptom trajectory over three measurements (latent slope)

| Region | Cluster Size | | Coordinates (center of mass) | | | Coordinates (at peak) | | | Mean F | SEM | Max Int | Post-hoc^a^ |
| --- | --- | --- | --- | --- | --- | --- | --- | --- | --- | --- | --- | --- |
|  | k | mm3 | CM RL | CM AP | CM IS | MI RL | MI AP | MI IS |  |  |  |  |
| Right Middle Temporal Gyrus | 93 | 1453.13 | -54.80 | 57.50 | 15.60 | -55.00 | 56.80 | 15.50 | 13.24 | 0.49 | 31.49 | -.62 |
| Right Rolandic Operculum, Right Insula Lobe | 85 | 1328.13 | -43.30 | 17.70 | 15.40 | -40.00 | 16.80 | 18.00 | 11.52 | 0.29 | 23.35 | -.62 |
| Right Postcentral Gyrus | 85 | 1328.13 | -30.50 | 31.90 | 54.10 | -27.50 | 31.80 | 58.00 | 13.38 | 0.46 | 30.34 | -.65 |
| Right SMA, Left SMA, Left Middle Cingulate Cortex | 83 | 1296.88 | -3.40 | 3.90 | 50.30 | -2.50 | 4.20 | 48.00 | 12.35 | 0.35 | 25.78 | -.64 |
| Left Rolandic Operculum, Left Insula Lobe | 72 | 1125.00 | 48.20 | -1.30 | 8.70 | 50.00 | -0.80 | 10.50 | 13.23 | 0.46 | 26.09 | -.66 |
| Right Rolandic Operculum, Right Superior Temporal Gyrus | 65 | 1015.63 | -56.40 | 3.20 | 6.90 | -52.50 | 4.20 | 5.50 | 13.58 | 0.55 | 27.80 | -.64 |
| Right Supramarginal Gyrus | 58 | 906.25 | -55.40 | 22.50 | 26.70 | -57.50 | 14.20 | 28.00 | 12.07 | 0.32 | 21.07 | -.61 |

*Note*. Cluster-corrected voxel-wise linear multivariate model results are presented here summarizing regions showing a significant intercept-by-condition interaction. Location lists regions in descending order based on proportion overlap with cluster. k=number of voxels in cluster, mm3=cluster volume, CM=center of mass of cluster, MI=max intensity (peak), SEM=standard error of the mean, LR=left-right (x), PA=posterior-anterior (y), IS=inferior-superior (z).

^a^Post-hocs are correlation coefficients between the latent slope and the % signal change in the task contrast.

*Neural Response to Error*

Table S7 details all significant results for the error analysis. Significant slope-by-error interactions were observed in four regions, the right fusiform gyrus (*F*(1,38)=28.68, *p*<.001, η^2^<.43, *r*(41)= -.63), the left middle frontal gyrus (*F*(1,38)=33.30, *p*<.001, η^2^<.47, *r*(41)=-.67), the left middle cingulate cortex (*F*(1,38)=35.23, *p*<.001, η^2^<.48, *r*(41)=-.67), and the left postcentral gyrus (*F*(1,38)=18.28, *p*<.001, η^2^<.33, *r*(41)=-.56). Another cluster was found overlapping with the left putamen, but upon removal of an outlier, the cluster was no longer significant and is therefore not presented here.

Table S7. Significant associations of neural activity during error processing with anxiety symptom trajectory over three measurements (latent slope).

| Region | Cluster Size | | Coordinates (Center of Mass) | | | Coordinates (At Peak) | | | Mean | SEM | Max Int | Post-hoc^a^ |
| --- | --- | --- | --- | --- | --- | --- | --- | --- | --- | --- | --- | --- |
|  | k | mm3 | CM RL | CM AP | CM IS | MI RL | MI AP | MI IS |  |  |  |  |
| Right Fusiform Gyrus, Right Interior Occipital Gyrus | 148 | 2313 | -33.7 | 68.8 | -10.8 | -32.5 | 86.8 | -9.5 | 13.02 | 0.27 | 24.22 | -.63 |
| Left Middle Frontal Gyrus | 117 | 1828 | 30.2 | -34.4 | 32.3 | 32.5 | -33.2 | 30.5 | 15.72 | 0.52 | 34.69 | -.67 |
| Left Middle Cingulate Cortex | 75 | 1172 | 11.9 | 28.5 | 42.6 | 15 | 31.8 | 45.5 | 12.07 | 0.28 | 17.74 | -.67 |
| Left Postcentral Gyrus | 66 | 1031 | 35 | 40.6 | 61.2 | 32.5 | 46.8 | 68 | 11.46 | 0.29 | 20.68 | -.56 |

*Note*. Cluster-corrected voxel-wise linear multivariate model results are presented here summarizing regions showing a significant intercept-by-condition interaction. Location lists regions in descending order based on proportion overlap with cluster. k=number of voxels in cluster, mm3=cluster volume, CM=center of mass of cluster, MI=max intensity (peak), SEM=standard error of the mean, LR=left-right (x), PA=posterior-anterior (y), IS=inferior-superior (z).

^a^Post-hocs are correlation coefficients between the latent slope and the % signal change in the task contrast.

References

Morales, S., Zeytinoglu, S., Lorenzo, N. E., Chronis-Tuscano, A., Degnan, K. A., Almas, A. N., Pine, D. S., & Fox, N. A. (2022). Which anxious adolescents were most affected by the COVID-19 pandemic? *Clinical Psychological Science*, *10*(6), 1044-1059.
